# Inhalational anaesthetic agent consumption within a multidisciplinary veterinary teaching hospital: an environmental audit

**DOI:** 10.1038/s41598-024-68157-5

**Published:** 2024-08-02

**Authors:** Dany Elzahaby, Alessandro Mirra, Olivier Louis Levionnois, Claudia Spadavecchia

**Affiliations:** Anaesthesiology and Pain Therapy Section, Department of Clinical Veterinary Medicine, Vetsuisse Faculty, University of Bern, Bern, Switzerland

**Keywords:** Anaesthesia, Sustainability, Veterinary, Environment, Climate, Inhalant, Environmental impact, Climate change

## Abstract

Inhalational anaesthetic agents are routinely used in veterinary anaesthesia practices, yet their consumption contributes significantly to greenhouse gas emissions and environmental impact. We conducted a 55-day observational study at a veterinary teaching hospital in Switzerland, monitoring isoflurane and sevoflurane consumption across small, equine and farm animal clinics and analysed the resulting environmental impact. Results revealed that in total, 9.36 L of isoflurane and 1.27 L of sevoflurane were used to anaesthetise 409 animals across 1,489 h. Consumption rates varied among species, with small and farm animals ranging between 8.7 and 13 mL/h, while equine anaesthesia exhibited a higher rate, 41.2 mL/h. Corresponding to 7.36 tonnes of carbon dioxide equivalent in total environmental emissions or between 2.4 and 31.3 kg of carbon dioxide equivalent per hour. Comparison to human anaesthesia settings showed comparable consumption rates to small animals, suggesting shared environmental implications, albeit on a smaller scale. This research highlights the importance of continued evaluation of veterinary anaesthesia practices to balance patient safety with environmental stewardship; potential mitigation strategies are explored and discussed.

## Introduction

Modern veterinary anaesthesia management routinely incorporates multimodal techniques referred to as balanced anaesthesia, which involves the use of different drugs, inhalational anaesthetic agents, intravenous infusions, as well as locoregional techniques^1^. This strategy aims to mitigate the adverse effects associated with the use of high proportions of individual drugs, whilst simultaneously increasing the likelihood of desired effects^2^. This is especially relevant to inhalational anaesthetic agents. When administered appropriately, they can provide a stable plane of anaesthesia and be rapidly eliminated even after prolonged duration of administration. However, they can have significant adverse effects when used at relatively high concentrations. Reducing the consumption of inhalational agents, such as isoflurane and sevoflurane, improves the overall quality of the anaesthetic as well as patient safety. This strategy is already strongly encouraged and widely used within veterinary anaesthesia^1,3^. Additionally, there has been a growing push in recent years to further reduce consumption of inhalational agents due to their accumulation in the atmosphere, which contributes to climate change^4,5^.

The World Health Organization recognises climate change as a critical global factor, posing a fundamental threat to human health^6^. Global annual temperatures continue to rise, prompting environmental committees to actively explore mitigation strategies aimed at reducing the increasing rates^7^. However, these mitigation strategies often overlook the healthcare sector due to its medical necessity^8–10^. Despite the lack of external pressure from environmental committees, there exists an inherent awareness that the healthcare industry contributes significantly to global carbon emissions^9^, as a result, there is growing environmental accountability amongst healthcare professionals including anaesthetists^11^.

It is imperative for anaesthetists to recognise the potential environmental impact stemming from the use of potent greenhouse gasses. This necessitates the collection and informative dissemination of data on inhalant consumption during general anaesthesia. To the best of the authors’ knowledge, there is currently no published literature quantifying the consumption of inhalant anaesthetics in a multidisciplinary veterinary hospital setting. Therefore, the aim of this study was to assess volatile anaesthetic agent consumption at an equine, farm, and small animal teaching hospital (Bern, Switzerland) over a specified period (55-days) and to evaluate the resulting environmental implications. Beyond shaping practices within individual veterinary hospitals, these findings can enhance institutional awareness on a broader scale.

## Materials and methods

### Study design and setting

A prospective observational study was performed by monitoring volatile anaesthetic agent consumption at an equine, farm, and small animal teaching hospital over a span of 55-days. The study was conducted across a period reflective of a relatively normal caseload for the hospital. Across the three clinics, anaesthesia residents, interns, technicians, and students, conducted general anaesthesia under the supervision of a diplomate of the European or American College of Veterinary Anaesthesia and Analgesia. For this study, personnel that performed general anaesthesia are simply referred to as anaesthetists. A total of 20 anaesthesia machines and 29 vaporisers (Supplementary S1) were used across the three clinics and anaesthesia was performed using circle rebreathing systems.

Anaesthetists at the small animal clinic had the option to choose between isoflurane or sevoflurane based on case suitability, whilst isoflurane was the only volatile anaesthetic agent used in the equine and farm clinics. The anaesthetists were responsible for adjusting the carrier gas mixture (composed of oxygen and medical air), as well as determining the appropriate fresh gas flow rates and vaporiser settings. Adjunct anaesthetic treatments to inhalational agents, such as loco-regional techniques or intravenous infusions, were also left to the discretion of the responsible anaesthetist based on clinical requirements. Prior to the beginning of the study all vaporizers were assigned a label used for tracking and reporting purposes.

### Inhalant gas consumption measurement

Total volatile anaesthetic agent consumption was measured daily within the equine and farm animal clinics, and weekly within the small animal clinic. This was achieved by weighing the isoflurane and sevoflurane bottles (Attane^M^, 250 mLs, Piramal Healthcare Ltd, India) used to fill all individually labelled vaporizers before and after they were filled. Vaporizers were filled consistently to the indicated full level by the same investigator. The weighing of bottles was performed using a precision balance (PCB 1000-2, Kern & Sohn GmbH, Balingen-Frommern, Germany; maximum weight: 1000 g; d: 0.01 g). The balance was calibrated initially by the manufacturer and then tested weekly using 1-, 5- and 20-g test weights to ensure conformity in results. The weights of the bottles were recorded both before and after filling, and the differences were subsequently calculated. The consumption of isoflurane and sevoflurane (in millilitres) were determined by dividing the weight difference by their respective densities, being 1.49 g/mL for isoflurane and 1.53 g/mL for sevoflurane^12^. In the event an operator was unavailable, theoretical volatile anaesthetic consumption was estimated using flow rate and vaporizer setting data collected from anaesthetic protocols. This estimation is based on the work of Biro^12^, who derived the saturated gas volumes for isoflurane and sevoflurane as 195 and 185 mL, respectively. At 5-min intervals of anaesthetic, as follows:

$${\text{Fluid}}\;{\text{Volatile}}\;{\text{Agent}}\;\left( {{\text{mL}}} \right) = \frac{{{\text{Mean}}\;{\text{Fresh}}\;{\text{Gas}}\;{\text{Flow}}\;\left( {{\text{mL}}/\min } \right) \times {\text{Mean}}\;{\text{Agent}}\;{\text{Conc}}.\;\left( {{\text{Vol}}\% } \right) \times {\text{Anaesthetic}}\;{\text{Duration}}}}{{{\text{Saturated}}\;{\text{Gas}}\;{\text{Volume}}\;\left( {{\text{mL}}} \right) \times 100 \, \left( {{\text{Vol}}\% } \right)}}.$$

Volatile anaesthetic consumption rates were determined for each species. To allow this calculation, each anaesthetic machine was equipped with a printed QR code, enabling anaesthetists to scan and complete a survey requesting various details following each anaesthetic event, including the duration of anaesthetic, patient details, amongst others outlined in detail in Supplementary S2. Responses from the survey were consolidated into Microsoft Excel. Duration of anaesthetic collected from uploaded data and the derived volume from manually weighed bottles allowed the calculation of rate of consumption (mL/h). Quality assurance measures were implemented through daily reviews to ensure all cases have been comprehensively logged by anaesthetists. In cases of missing submissions, protocols were retrieved from hospital records.

### Environmental impact measurement

For environmental impact assessment, the carbon dioxide equivalent (CO_2_e) of measured volatile agent totals was determined. This calculation was based on the established environmental properties of these gases. The Global Warming Potential over a 100-year period (GWP_100_) is a metric for a gas’s greenhouse potential and was used in this study for comparative analysis, with GWP_100_ being 510 and 130 for isoflurane and sevoflurane, respectively^4^. GWP_100_ and the weight of inhalant measured during the study period were used to calculate CO_2_e in tonnes (t) using the following formula:

$${\text{CO}}_{2} {\text{e}}\;\left( {\text{t}} \right) = {\text{Weight}}\;{\text{of}}\;{\text{Gas}}\;\left( {\text{t}} \right) \times {\text{GWP}}_{100} .$$

Isoflurane and sevoflurane CO_2_e totals were calculated for the small animal, equine and farm animal clinics over the study period as well as their respective averages per hour of anaesthesia. Additionally, carbon dioxide emissions were compared to car mileage for comparability and reader relatability. Car mileage equivalent was calculated assuming European average petrol passenger vehicle emissions are approximately 134 g of CO_2_e per kilometre driven^14^.

## Results

### Total isoflurane/sevoflurane consumption

A total of 409 patients had undergone inhalational anaesthesia during the study period (Table 1). Weighing of isoflurane and sevoflurane accounted for 402 of these cases, however 7 cases did not have corresponding inhalant weight measurements due to investigator availability. Data corresponding to these cases were estimated by calculating theoretical inhalant consumption using vaporizer settings and flow rate data derived from anaesthetic protocols. Hence, in total, 9.36 L of isoflurane and 1.27 L of sevoflurane were consumed.

**Table 1:** This table summarizes the number of patients anaesthetised with isoflurane or sevoflurane across small animal, equine and farm clinics during the study period.

	Small animal		Equine	Farm
	Isoflurane	Sevoflurane	Isoflurane	Isoflurane
Total patients	303	54	35	17
Total inhalant (L)	5.90	1.27	2.8	0.66
Total duration (min)	40,736	6153	4080	3040
Average weight (kg)	14.91	12.89	416.37	195.01
Average inhalant per patient (mL/patient)	19.47	23.52	80	38.82
Average consumption (mL/h)	8.7	12.4	41.2	13

It includes total anaesthetic inhalant volume per patient group, duration of use, average patient weight, and calculations for average inhalant per patient (mL/patient) and average consumption (mL/h), based on total volume and duration.

### QR code submissions

Overall, 380 out of 409 cases were submitted through the QR scanning system. 29 cases omitted submissions, resulting in a 92.9% completion rate. Patient data including weight, vaporizer used, patient identification and anaesthetic duration for these cases were retrospectively supplemented following retrieval from hospital records. Patient data and resulting volatile anaesthetic consumption are presented in Table 1.

### Environmental impact of measured consumption

In total, 409 cases across all species within the three clinics contributed 7.36 t of CO_2_e via inhalant gas emissions alone (Table 2). Isoflurane and sevoflurane emitted from the small animal clinic corresponded to 4.48 and 0.25 t of CO_2_e, respectively. The equine and farm animal clinics were responsible for 2.13 t and 0.5 t of CO_2_e, respectively. For reader relatability, car mileage comparisons can be viewed in Table 2.

**Table 2:** Environmental impact of inhalant agent consumption corresponding to the small animal, farm and equine clinics shown as carbon dioxide equivalent (CO_2_e) in tonnes (t).

	Small animal		Equine	Farm
	Isoflurane	Sevoflurane	Isoflurane	Isoflurane
Total CO_2_e (t)	4.48	0.25	2.13	0.50
Average consumption (kg of CO_2_e/h)	6.6	2.4	31.3	9.87
Total equivalent in kilometres travelled	33,433	1866	15,896	3731
Kilometres driven per one hour of anaesthetic	49	18	234	74

Total CO_2_e and total duration were used to calculate average consumption in kg of CO_2_e/h. Environmental totals were compared to car mileage for general reader relatability.

## Discussion

Overall, the three clinics collectively emitted 7.36 t of CO_2_e through inhalant gas emissions over a 55-day period. Extrapolating this figure over one year, assuming a consistent caseload, yields an estimated emission of approximately 49 t of CO_2_e, or 364,509 miles driven^14^. This contribution, solely attributed to volatile anaesthetic emission, underscores its notable environmental impact. In this study, isoflurane consumption rates were 8.7 mL/h, 41.2 mL/h and 13 mL/h in the small animal, equine and farm animal clinics, respectively, while sevoflurane consumption was 12.4 mL/h in the small animal clinic. Additionally, sevoflurane was found to have a lower CO_2_e per hour when compared to isoflurane in the small animal clinic. Although veterinary literature on this topic is limited, these values can be contextualised by comparing to their equivalent consumption rates in human anaesthesia settings. Vithayathil et al.^15^ showed that low-flow anaesthesia techniques, combined with different anaesthetic protocols, resulted in isoflurane consumption rates ranging between 8.9 to 12.7 mL/h. These rates are similar to those observed in this study within the small and farm animal clinics (8.7–13 mL/h), while the equine consumption rates exceeded this range by a minimum of threefold. In terms of sevoflurane, Singaravelu and Barclay^16^ reported a mean consumption rate of 14 mL/h, also similar to the rate recorded here (12.4 mL/h).

During equine anaesthesia, consumption rates were relatively higher, attributable to several factors that hinder the ability to implement low-flow techniques. Low flow involves the implementation of semi-closed or closed rebreathing circuits, aiming for low flow rates (0.5–1 L/min) or minimal flow rates (0.25–0.5 L/min)^5^. Unlike in human and small animal anaesthesia, achieving low flow rates is often unattainable in equine anaesthesia settings. In anaesthetised horses, the metabolic rate is estimated to be between 1.78 to 1.89 mL/kg/min^17^. Thus, a 500 kg horse would necessitate between 890 to 945 mL/min of oxygen under anaesthesia, requiring a higher supply to prevent hypoxaemia. Additionally, equine breathing circuits encompass a significantly larger volume, approximately 7 times greater than small animal circuits. The result is a relatively greater time constant when referring to delivery of anaesthetic agents. Thereby, relatively higher fresh gas flow rates and vaporizer settings are required to mitigate the larger size of the anaesthetic circuit to ensure rapid uptake of anaesthetic agent to the animal, at least in the initial period. Furthermore, while the research on the subject is limited, the potential accumulation of pulmonary methane in herbivores during low-flow anaesthesia raises concerns about its safety and advisability^18^. As such, low flow techniques are not generally applied in equine anaesthesia, resulting in significantly higher consumption rates compared to small and farm animals.

The results of this study show that the actual consumption rates in small animals exceed the expected potential consumption if ideal low-flow rates were utilized. Using an anaesthetic impact calculator, it was determined that when using isoflurane at minimum alveolar concentration (MAC) and 1 L/min (low-flow), 3 and 3.7 kg of CO_2_e are emitted per hour in dogs and cats, respectively^19^. Similarly, sevoflurane consumption rates under identical conditions resulted in 1 and 1.7 kg of CO_2_e per hour in dogs and cats, respectively^19^. However, our study revealed higher consumption rates, with an average of 6.6 kg of CO_2_e per hour using isoflurane and 2.4 kg of CO_2_e per hour using sevoflurane for small animal patients (comprised of 71% dogs, and 29% cats). Potential explanations for this discrepancy could be that anaesthetists at this facility tend to use higher flow rates than 1 L/min or maintain higher vaporizer settings compared to MAC. However, the hospital’s implementation of balanced anaesthesia protocols, incorporating intravenous infusions and loco-regional techniques, aims to achieve an average inhalant concentration similar, if not lower than, MAC. Hence, it seems more plausible that the observed disparity could be attributed to the flow rates exceeding 1L/min, warranting an assessment of flow-rate management within this hospital setting. It is also important to acknowledge that these findings reflect a more realistic anaesthetic scenario, characterized by common practices employing higher flow rates and vaporizer settings to accelerate anaesthetic uptake during the early post-induction period.

Environmental sustainability in the human medical field has prompted discussions on the replacement of inhalant agents with total intravenous anaesthesia (TIVA). Propofol TIVA produces notably lower greenhouse gas emissions compared to either isoflurane or sevoflurane, even when factoring in the disposable material required for intravenous administration^20,21^. TIVA has been safely implemented in dogs^22,23^ and cats^24,25^ for select procedures, with studies demonstrating its effectiveness in achieving similar clinical outcomes compared to inhalational anaesthesia. However, longer infusion times using propofol or alfaxalone TIVA have been associated with prolonged recovery in both dogs and cats, due to drug accumulation and longer elimination half-time^26^. Feline patients, in particular, can experience extended recovery periods due to their slower metabolism of propofol, which is hypothesized to be due to a deficiency in hepatic glucoronyl transferase, an enzyme required in glucuronidation^27^.

In horses, prolonged durations of TIVA can compromise the quality of the recovery period^28,29^. Notably, the American College of Veterinary Anaesthesia and Analgesia advises restricting the use of TIVA in horses to anaesthetic procedures expected to be less than 60 min in duration^28^, limiting its use to relatively non-invasive and shorter duration procedures. For more invasive procedures such as a complicated fracture repair or colic-related abdominal surgery, a balanced protocol including inhalant agents is typically recommended. Given this reliance on inhalant anaesthesia, it highlights the importance of evaluating strategies for integrating TIVA rather than pursuing complete elimination of inhalants; an approach referred to as partial intravenous anaesthesia (PIVA).

It is also important to note that while propofol offers advantages in terms of reduced greenhouse gas emissions compared to gas anaesthesia, it also poses negative downstream environmental risks often overlooked in such comparisons. Propofol is non-biodegradable and highly toxic to aquatic organisms^30,31^. Considering propofol waste within human operating rooms can be as high as 45%, improper disposal in general waste or into sewage can have detrimental ecological effects^31^. Further studies are required to evaluate the extent of the ecological impact and potential mitigation benefits associated with the disposal of propofol waste.

Gas capture devices have been traditionally used in place of active or passive scavenging capabilities. Gas capture canisters using various gas adsorbent technology such as activated charcoal, can be used to prevent anaesthetic gas emissions from being scavenged into the environment^32^. While they successfully minimize occupational exposure within the workplace, their environmental benefit is limited. These canisters are typically disposed of in general landfills after use, where the saturated charcoal desorbs over time, releasing anaesthetic agents into the environment and posing similar environmental risks to traditional scavenging methods^21^.

Gas capture and recycling technology presents a promising alternative to conventional scavenging methods in combating inhalant emissions, with the potential to enhance anaesthetic gas sustainability and reduce environmental pollution associated with inhalant anaesthesia. However, comprehensive lifecycle assessments are crucial to evaluate its overall environmental sustainability, which are challenging to ascertain given the proprietary and confidential nature of the technology involved. Moreover, there is significant room for improvement in recycling practices. Despite the effectiveness of gas capture devices like CONTRAFluran (ZeoSys Medical Gmbh, Luckenwalde, Germany) in scavenging gases^33^, Hinterberg et al.^34^ showed that these devices only captured 25% of administered desflurane. This highlights the issue of post-anaesthetic gas emissions, which bypass capture and recycling. Additionally, regulatory approval from governmental bodies is necessary to authorise the use of recycled agents in animals, thereby posing an additional barrier to this method’s implementation. Although in its infancy, this method shows considerable promise.

A limitation of this study was the measurement method used to estimate gas consumption. In typical practice, vaporizers are not filled as frequently as they were during the scope of this study, leading to unavoidable evaporation of inhalant agent, albeit in minute amounts. Moreover, the methodology relied on the accuracy of the precision scale. However, in comparison to previously used methods, the methodology used here was deemed to provide a more accurate representation of consumption. In validating the method used here, we compared the bottle weighing method to the theoretical calculation method typically used in other studies^13^. We compared 10 sample cases using both methods and found that at least a 12% discrepancy exists between measurements, primarily attributable to record-keeping errors. Consequently, given the theoretical calculation method’s high sensitivity to the quality of record-keeping, a significant potential for human error and subsequent inaccuracies in outcomes becomes inevitable, thereby inferring more accurate results using the bottle weighing method. Nevertheless, the use of the theoretical calculation was unavoidable in 7 out of the 409 cases analysed (1.7%), which could potentially introduce discrepancies in the results. Additionally, a potential source of bias in the results could be attributed to variations in flow rate settings, which were likely influenced by individual experience level. Furthermore, the study’s duration was limited due to the extensive resources required for its implementation, making longer timescales prohibitive. Although, this limitation could introduce bias (especially in smaller species samples), the results represented here are not reliant on statistical inference but rather focused on observational data, rendering this limitation less relevant in this context.

## Conclusion

This study provides insight into the significant gas emissions resulting from inhalant anaesthesia practices at a multidisciplinary veterinary hospital and the consequential environmental impact. Further, it highlights that environmental contributions differ based on the species being anaesthetised, with horse anaesthesia having the most significant impact on average. Additionally, sevoflurane should be considered more frequently due to its preferable environmental properties compared to those of isoflurane. The comparison of consumption rates and hypothetical low-flow anaesthesia suggests a need for the introduction of training seminars. It also emphasises the need for continued evaluation of anaesthetic practices to minimize emissions, while ensuring patient-safety. As inhalant use in veterinary anaesthesia is presently unavoidable, exploration of alternative techniques and technologies is warranted to mitigate their adverse environmental contributions.

### Supplementary Information

Supplementary Information 1.

Supplementary Information 2.

## Data Availability

Data is made available upon reasonable request from authors.

## References

[1] Steffey, E. P., Mama, K. R. & Brosnan, R. J. Inhalation anesthetics. In *Veterinary Anesthesia and Analgesia* (eds Grimm, K. A. *et al.*) 297–331 (Wiley, 2015).

[2] Brown, E. N., Pavone, K. J. & Naranjo, M. Multimodal general anesthesia: Theory and practice. *Anesth. Analg.* **127**, 1246–1258 (2018). 30252709 10.1213/ANE.0000000000003668 PMC6203428

[3] Clutton, R. E. An anglocentric history of anaesthetics and analgesics in the refinement of animal experiments. *Animals* **10**, 1933 (2020). 33096686 10.3390/ani10101933 PMC7589666

[4] Vollmer, M. K. *et al.* Modern inhalation anesthetics: Potent greenhouse gases in the global atmosphere. *Geophys. Res. Lett.* **42**, 1606–1611 (2015). 10.1002/2014GL062785

[5] Jones, R. S. & West, E. Environmental sustainability in veterinary anaesthesia. *Vet. Anaesth. Analg.* **46**, 409–420 (2019). 31202620 10.1016/j.vaa.2018.12.008

[6] WHO. *Climate Change*. https://www.who.int/health-topics/climate-change (2024).

[7] Intergovernmental Panel on Climate Change (Ipcc). *Climate Change 2022—Impacts, Adaptation and Vulnerability: Working Group II Contribution to the Sixth Assessment Report of the Intergovernmental Panel on Climate Change* (Cambridge University Press, 2023). 10.1017/9781009325844.

[8] Charlesworth, M. & Swinton, F. Anaesthetic gases, climate change, and sustainable practice. *Lancet Planet. Health* **1**, e216–e217 (2017). 29851604 10.1016/S2542-5196(17)30040-2

[9] Pichler, P.-P., Jaccard, I. S., Weisz, U. & Weisz, H. International comparison of health care carbon footprints. *Environ. Res. Lett.* **14**, 064004 (2019). 10.1088/1748-9326/ab19e1

[10] Watts, N. *et al.* Health and climate change: Policy responses to protect public health. *The Lancet* **386**, 1861–1914 (2015). 10.1016/S0140-6736(15)60854-6 26111439

[11] England, N. H. S. & Improvement, N. Delivering a ‘net zero’national health service. *Lond. NHS Engl. NHS Improv.* (2020).

[12] Biro, P. Calculation of volatile anaesthetics consumption from agent concentration and fresh gas flow: Volatile anaesthetics consumption. *Acta Anaesthesiol. Scand.* **58**, 968–972 (2014). 25060161 10.1111/aas.12374

[13] McMillan, M. Sustainable veterinary anaesthesia: Single centre audit of oxygen and inhaled anaesthetic consumption and comparisons to a hypothetical model. *J. Small Anim. Pract.* **62**, 420–427 (2021). 33939176 10.1111/jsap.13316

[14] European Environment Agency. *Monitoring of CO*_*2*_* Emissions from Passenger Cars, 2022: Final data. EEA Geospatial Data Catalogue*. https://sdi.eea.europa.eu/catalogue/srv/api/records/992616f8-158f-4ecc-b978-814b81629db6.

[15] Vithayathil, R., Savitha, K. S., Dixit, N. & John, L. Target-controlled inhalational anesthesia-isoflurane consumption with adequacy of anesthesia monitoring in conventional and multimodal analgesia: A comparative study. *Anesth. Essays Res.* **16**, 143 (2022). 36249130 10.4103/aer.aer_43_22 PMC9558675

[16] Singaravelu, S. & Barclay, P. Automated control of end-tidal inhalation anaesthetic concentration using the GE Aisys Carestation™ †. *Br. J. Anaesth.* **110**, 561–566 (2013). 23293274 10.1093/bja/aes464

[17] Hubbell, J. A. E. & Muir, W. W. Oxygenation, oxygen delivery and anaesthesia in the horse. *Equine Vet. J.* **47**, 25–35 (2015). 24612155 10.1111/evj.12258

[18] Moens, Y., Gootjes, P. & Lagerweij, E. The influence of methane on the infrared measurement of halothane in the horse. *J. Vet. Anaesth.* **18**, 4–7 (1991). 10.1111/j.1467-2995.1991.tb00004.x

[19] Pierce, J. T. *Anaeshtetic Gases Calculator* (Association of Anaesthetists, 2015). https://anaesthetists.org/Home/Resources-publications/Environment/Guide-to-green-anaesthesia/Anaesthetic-gases-calculator.

[20] Ryan, S. & Sherman, J. Sustainable anesthesia. *Anesth. Analg.* **114**, 921–923 (2012). 22523405 10.1213/ANE.0b013e31824fcea6

[21] Sherman, J., Le, C., Lamers, V. & Eckelman, M. Life cycle greenhouse gas emissions of anesthetic drugs. *Anesth. Analg.* **114**, 1086–1090 (2012). 22492186 10.1213/ANE.0b013e31824f6940

[22] Suarez, M. A., Dzikiti, B. T., Stegmann, F. G. & Hartman, M. Comparison of alfaxalone and propofol administered as total intravenous anaesthesia for ovariohysterectomy in dogs. *Vet. Anaesth. Analg.* **39**, 236–244 (2012). 22405473 10.1111/j.1467-2995.2011.00700.x

[23] Tsai, Y.-C., Wang, L.-Y. & Yeh, L.-S. Clinical comparison of recovery from total intravenous anesthesia with propofol and inhalation anesthesia with isoflurane in dogs. *J. Vet. Med. Sci.* **69**, 1179–1182 (2007). 18057835 10.1292/jvms.69.1179

[24] Ilkiw, J. E. & Pascoe, P. J. Cardiovascular effects of propofol alone and in combination with ketamine for total intravenous anesthesia in cats. *Am. J. Vet. Res.* **64**, 913–917 (2003). 12856778 10.2460/ajvr.2003.64.913

[25] Mendes, G. M. & Selmi, A. L. Use of a combination of propofol and fentanyl, alfentanil, or sufentanil for total intravenous anesthesia in cats. *J. Am. Vet. Med. Assoc.* **223**, 1608–1613 (2003). 14664447 10.2460/javma.2003.223.1608

[26] Absalamon, A. R. (ed.) *Total Intravenous Anesthesia and Target Controlled Infusions: A Comprehensive Global Anthology* (Springer International Publishing, 2017).

[27] Pascoe, P. J., Ilkiw, J. E. & Frischmeyer, K. J. The effect of the duration of propofol administration on recovery from anesthesia in cats. *Vet. Anaesth. Analg.* **33**, 2–7 (2006). 16412126 10.1111/j.1467-2995.2005.00216.x

[28] Hubbell, J. A review of the American College of Veterinary Anesthesiologists guidelines for anesthesia of horses. *Proc. Am. Equine Pr.* **54**, 48–53 (2008).

[29] Wagner, A. E. Complications in equine anesthesia. *Vet. Clin. North Am. Equine Pract.* **24**, 735–752 (2008). 19203709 10.1016/j.cveq.2008.10.002

[30] Sherman, J. D. & Barrick, B. Total intravenous anesthetic versus inhaled anesthetic: Pick your poison. *Anesth Analg.* **128**, 13–15 (2019). 30550470 10.1213/ANE.0000000000003898

[31] Mankes, R. F. Propofol wastage in anesthesia. *Anesth. Analg.* **114**, 1091–1092 (2012). 22415537 10.1213/ANE.0b013e31824ea491

[32] Birgenheier, N., Stoker, R., Westenskow, D. & Orr, J. Activated charcoal effectively removes inhaled anesthetics from modern anesthesia machines. *Anesth. Analg.* **112**, 1363–1370 (2011). 21543783 10.1213/ANE.0b013e318213fad7

[33] Dubernet, M. *et al.* Using the anesthetic gas filter CONTRAfluran while on cardiopulmonary bypass: Preliminary study of the feasibility, security, and efficiency. *J. Cardiothorac. Vasc. Anesth.*10.1053/j.jvca.2023.11.009 (2023). 38061918 10.1053/j.jvca.2023.11.009

[34] Hinterberg, J. *et al.* Efficiency of inhaled anaesthetic recapture in clinical practice. *Br. J. Anaesth.* **129**, e79–e81 (2022). 35589427 10.1016/j.bja.2022.04.009

