# Supplementary material for: Inhalational anaesthetic agent consumption within a multidisciplinary veterinary teaching hospital: an environmental audit
Source: Sci Rep. 2024 Aug 2;14:17973. doi: 10.1038/s41598-024-68157-5 (PMC11297182; doi:10.1038/s41598-024-68157-5)
Supplement: Supplementary file 1 — Supplementary Information 1. [file 41598_2024_68157_MOESM1_ESM.pdf]

Supplementary S1

This table details the inventory of various vaporizer types used in the small animal, equine, and farm animal clinics, specifying the total quantities and the corresponding filling techniques (pour and key techniques).

VAPORIZER TYPE	*Small Animal*	*Equine*	*Farm*	*Pour Technique*	*Key Technique*
Tec 850 Iso	3			✓	
Tec 7 Iso	8	1		✓	
Tec 7 Sevo	5			✓	
Ohmeda Isotec 5	2		1		✓
Ohmeda Sevotec 5	2				✓
Tec 3 Iso		2	1		✓
Draeger Vapor 2000 Iso	3			✓	
Draeger Vapor 2000 Sevo	1			✓	
