# Supplementary material for: Inhalational anaesthetic agent consumption within a multidisciplinary veterinary teaching hospital: an environmental audit
Source: Sci Rep. 2024 Aug 2;14:17973. doi: 10.1038/s41598-024-68157-5 (PMC11297182; doi:10.1038/s41598-024-68157-5)
Supplement: Supplementary file 2 — Supplementary Information 2. [file 41598_2024_68157_MOESM2_ESM.pdf]

Supplementary S2

Below is the online questionnaire that anaesthetists were asked to complete following an anaesthetic.

Anaesthesia Questionnaire

Please complete this questionnaire at your earliest leisure ie. preferably before the day starts tomorrow!
You may complete a single questionnaire even if multiple vaporisers have been used.

[redacted] Switch account

Not shared

* Indicates required question

Date of anaesthesia

MM DD YYYY

__ / __ / ____

Time of day *

- ◯ 8 am to 6 pm
- ◯ 6 pm to 8 am

Please fill in the patient ID *

Your answer

How much does the patient weigh in kgs? *

Your answer

Which vaporizer did you use for this procedure? *

Choose ▾

How long did you use this this vaporizer in minutes? *

Your answer

Which description fits your role? *

- ◯ MTA
- ◯ VAT

- TPA
- Doctorand
- Anaesthesia Resident
- Intern
- Diplomat
- Student
- Other: ____________

Did you need to fill the vaporiser during this procedure?

- Yes
- No

Any comments that you think may be relevant

Your answer

ONLY FILL BELOW IF YOU HAVE USED MORE THAN ONE VAPORIZER

If this is not the case, please do not answer the

Which other vaporiser did you use during this anaesthesia?

Choose

How long did you use this vaporiser for in minutes?

Your answer

Which other vaporiser did you use during this anaesthesia?

Choose

How long did you use this vaporiser for in minutes?

Your answer

Choose

How long did you use this vaporizer for in minutes?

Your answer

Thank you sincerely for taking the time to help me complete this study.
If you have any questions, please feel free to contact me at:

[redacted]

Submit

Clear form

Never submit passwords through Google Forms.

This content is neither created nor endorsed by Google. Report Abuse - Terms of Service - Privacy Policy

Google Forms
